# Team experiences of the root cause analysis process after a sentinel event: a qualitative case study

**DOI:** 10.1186/s12913-023-10178-3

**Published:** 2023-11-08

**Authors:** Silje Liepelt, Hildegunn Sundal, Ralf Kirchhoff

**Affiliations:** 1 Department of Health Sciences, Ålesund, Faculty of Medicine and Health Sciences, Norwegian University of Science and Technology, Larsgårdsvegen 2, Ålesund, 6025 Norway; 2 Faculty of Health Sciences and Social Care, Molde University College, PO. Box 2110, Molde, 6402 Norway

**Keywords:** Root cause analysis, Qualitative case study, Sentinel events, Organizational learning, Norway, Childbirth

## Abstract

**Background:**

Root cause analysis (RCA) is a systematic approach, typically involving several stages, used in healthcare to identify the underlying causes of a medical error or sentinel event. This study focuses on how members of a Norwegian RCA team experience aspects of an RCA process and whether it complies with the Norwegian RCA method.

**Method:**

Based on a sentinel event in which a child died unexpectedly during childbirth in a Norwegian hospital in 2021, the following research questions are addressed: 1. What was the RCA team’s experience of the RCA process? 2. Was there compliance with the Norwegian RCA method in this case? A case study was chosen out of the desire to understand complex social phenomena and to allow in-depth focus on a case.

**Results:**

The result covered three main themes. The first theme related to the hospital’s management system and aspects of the case that made it challenging to follow all recommendations in the Norwegian RCA guidelines. The second theme encompassed external and internal assessment. The RCA team was composed of members with methodological and medical expertise. However, the police’s involvement in the case made it complex for the team to carry out the process. The third and final theme covered intrapersonal challenges RCA team members faced. Team members experienced various challenges during the RCA process, including being neutral, dealing with role-related challenges, grappling with ambivalence, and managing the additional time burden and resource constraints. As anticipated in the RCA guidelines, the team’s ability to remain neutral was tested.

**Conclusion:**

The findings of this study can help stakeholders better comprehend how an inter-professional RCA teamwork intervention can affect a healthcare organization and enhance the teamwork experience of healthcare staff while facilitating improvements in work processes and patient safety. Additionally, these results can guide stakeholders in creating, executing, utilizing, and educating others about RCA processes.

## Introduction

In the healthcare landscape, the paramount objective has always been to ensure the safety and well-being of patients. Patient safety, defined as “the absence of preventable harm to a patient and reduction of risk of unnecessary harm associated with healthcare to an acceptable minimum” [1], is the bedrock of his commitment. James Reason's seminal research has been instrumental in shaping our understanding of patient safety in healthcare [2]. Reason's pioneering work established the fundamental principles of the pivotal safety paradigm called "Safety I", while the "Safety II" paradigm is closely associated with researchers E. Hollnagel and J. Braithwaite [3]. “Safety I” [3] primarily focuses on reducing the number of adverse outcomes by identifying and addressing the causes of errors and hazards, essentially striving to prevent things from going wrong. In contrast, Safety II is characterized by a proactive approach that emphasizes the resilience and adaptability of healthcare systems to success under various conditions, aiming to ensure that things go right rather than solely preventing them from going wrong. Understanding these paradigms is vital in contemporary patient safety efforts, and they provide a framework for exploring the complexities of human error and system resilience in healthcare.

Root cause analysis (RCA), a pivotal method within patient safety practices, closely aligns with Safety I [4]. RCA provides a structured and systematic approach for investigating sentinel events, which are unexpected occurrences resulting in patient death, serious physical or psychological injury, or a risk thereof [5, 6]. The term “sentinel” implies that the event may be a warning sign of ongoing problems in the process of care that may lead to similar events in the future. Such events are not only debilitating to patients and their next of kin; they can also impact the livelihood of healthcare providers [7], who may become second victims of the error [8].

Despite the recognized importance of RCA in patient safety [9, 10], a notable knowledge gap exists in understanding the practical implementation of RCA within healthcare settings [11]. This paper aims to bridge this gap by embarking on an analysis of the experience of an RCA team investigation of a sentinel event, specifically the unexpected death of a child during childbirth. We seek to determine whether the team adhered to the recommended guidelines for conducting an RCA, shedding light on the effectiveness of the RCA process in the context of this event. By addressing this knowledge gap, our study contributes to the broader goal of enhancing patient safety practices and hopefully mitigating the risk of similar events.

In this paper, we analyze the experience of an RCA team process that investigated a sentinel event in which a child died unexpectedly during childbirth and determine whether the team followed the recommendations outlined in the RCA guidelines. First, we describe RCA and its place in the Norwegian health service. Next, we describe this study’s research design and data. Then, in the methodology section, we detail how we employ the case study method to explore our research questions. Finally, we present our results followed by a discussion of their implications and limitations and suggestions for future research.

### Root cause analysis

RCA is an umbrella term for methodologies and tools for the retrospective and structured investigation of adverse incidents, near misses, and sentinel events [12, 13]. RCA was first formally introduced into healthcare in 1996 by The Joint Commission for Accreditation of Healthcare Organizations [14]. The method is designed to identify the factors that underlie a sentinel event and is the most often used form of comprehensive systematic analysis [6]. Organizations worldwide use RCA to identify underlying causes of adverse events and near misses to uncover factors that lead patient safety to be compromised [13]. It is a reactive type of accident analysis and a blended approach combining other methodologies and instruments for investigating adverse events, causalities, and tragic events. RCA relies on the premise that accurate and analytic processes can identify hazards and help avoid adverse events.

A decision-making employee (process owner) can initiate an RCA process. The first step in an RCA is forming a multidisciplinary team to analyze and define the problem [7]. The RCA involves a team of healthcare professionals from various disciplines, such as physicians, nurses, administrators, and quality improvement experts [6]. The team approach is essential for identifying the underlying causes of sentinel events. The RCA team examines the event, gathers data, and analyzes information to identify the root causes and contributing factors. The extent to which RCA is used may vary by country and healthcare system.

Many hospitals use RCA as their primary investigative method [9]. RCA use in the medical field has been limited [7], and the method has been applied widely without sufficient attention paid to what makes it work in various contexts and without adequate customization for the specifics of healthcare [13, 15]. Despite the widespread implementation of RCA, authors argue that RCA may not reduce the risk of recurrence as intended [15–20]. According to Percarpio et al. [15], there is anecdotal evidence that RCA effectively improves patient safety.

There is a paucity of literature on how the process of RCA can be implemented effectively [21]. Some studies have examined the types of cases for which RCAs have been performed [16]. Case studies could support shared knowledge and provide benchmarks for improving the RCA method [15] and be a powerful tool in supporting improvements in the use of RCA in healthcare. Clinician participation in RCA is vital, as these initiatives recognize and address essential aspects of patient care [7]. To better support evidence-based practice, research should include detailed descriptions of how team members experience the process and what insights they gain from it.

### The Norwegian regulatory regime for qualitative improvement and RCA

Several governmental initiatives have been launched in Norway in recent decades to facilitate hospitals’ continuous attention to patient safety and to increase the overall quality of their healthcare services [22]. The Norwegian National Action Plan for Quality and Patient Safety (2019–2023) focuses on structural and cultural dimensions of safety improvements [23]. In 2017, The Norwegian healthcare system implemented a regulatory framework to support local quality and safety efforts in hospitals [22]. “Leading the way to zero” [6] initiatives entered the Norwegian healthcare system in 2019, having been introduced in the Directorate of Health’s National Action Plan for Patient Safety and Quality Improvement (2019–2023) [23]. The mission was to improve the quality and safety of healthcare and to eliminate patient harm by implementing reliable processes in healthcare. The plan highlights the importance of investigating failures in healthcare so that organizations can understand why errors occurred and work to prevent similar mistakes in the future. In Norway, hospital organizations must ensure their employees have relevant competence and training. Current management and training programs include learning about quality improvement methods and systematics [23, 24].

The initial reporting system in Norway, mandated by legislative requirements, was established following a parliamentary decision in the early 1990s. This decision was founded in a Norwegian government proposal to the Parliament, known as Ot.prp.nr 33 (1991–1992) [25]. These legislative alterations led to amendments in the law pertaining to hospitals, representing an important milestone in the country`s healthcare reporting and oversight framework. The current reporting system for serious adverse events in healthcare services was officially established in Norway in 2010. This was an important change in healthcare legislation to improve patient safety and learn from mistakes and incidents in the healthcare system. The reporting requirement is integral to the regulatory framework designed to ensure patient safety and maintain high-quality standards within the Norwegian healthcare system.

**Fig. 1:**
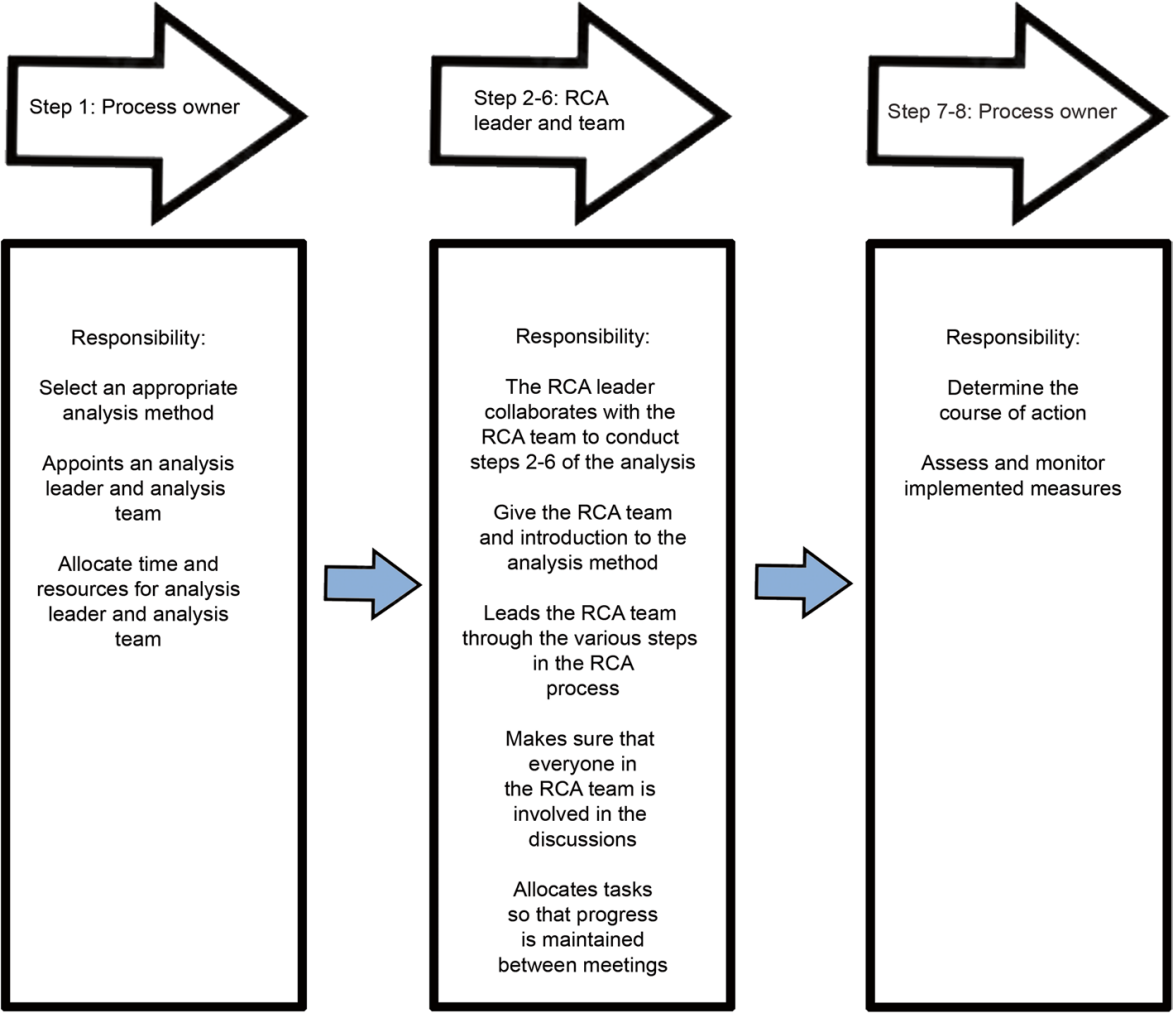
Roles and responsibilities in the Norwegian RCA process

The Norwegian RCA guidelines, inspired by the work of Bagian [19] at the US Department of Veterans Affairs (VA) [4], was published in 2016 to provide methodological support for the improvement of patient safety [4]. The guideline essentially represents an adapted version of the publication initially released by the Swedish Association of Local Authorities and Regions [26]. The methodology identifies eight stages of the RCA process: 1) initiating analysis, 2) gathering facts, 3) describing the course of events, 4) identifying underlying causes, 5) measures and methods for follow-up, 6) writing a final report, 7) deciding on measures, and 8) evaluation and follow-up measures (Fig. 1). It involves different stakeholders performing different tasks using the methodology while relating to organizational conditions, rules, and guidelines within a physical environment. Our study focuses primarily on steps 2–6 (see Fig. 1) to investigate how members of an RCA team experience these aspects of an RCA process. We also explore whether there is compliance in practice with the Norwegian RCA method. Based on a sentinel event in which a child died unexpectedly during childbirth in a Norwegian hospital in 2021, we address the following research questions: 1. What was the RCA team’s experience of the RCA process? 2. Was there compliance with the Norwegian RCA method in this case?

## Methodology

### Design

The case study design is appropriate when study outcomes relate to clinical practice [27]. A case study was chosen out of the desire to understand a complex social phenomenon [28], to allow in-depth focus on a single case, and to retain a holistic and real-world perspective in studying the RCA team members’ description of behavior, organization, and managerial learning processes. A case study is a flexible research design that captures holistic and meaningful characteristics of actual life events [28]. Case studies can provide a detailed understanding of what is happening and solid grounds for improvement [29]. Case study research has a strong advantage in examining the relevant process [28]. It can capture the complexity of a case, including appropriate changes over time, and attend fully to contextual conditions, including those that potentially interact with the case. However, within the evaluation field, case study research can perform a precious additional function in explaining how the “case,” usually a planned intervention or an ongoing initiative, works [28].

In this study, we used the Norwegian national RCA guidelines [4] to develop the interview guide. To answer the research questions, we used repeat interviews, also called follow-up interviews [30], that allow both the interviewer and the participants to reflect on what was discussed in the first interview, allowing new insights or aspects to emerge. These interviews were used to further explore and clarify topics discussed in earlier interviews and to gather additional information. We conducted the transcription and a preliminary thematic analysis before we interviewed the participants for the second time. This meant we could pursue topics we thought were interesting to follow up on and make thick descriptions [31] to ensure the findings were transferable between the research team and the participants we studied.

Interview data and relevant documents (Table 1) were collected and analyzed in separate phases of the research process. We identified patterns in the data material and extracted information and standard features to create an overall impression of the completed RCA process. Phase one explores the RCA team participants’ experiences with the pre-work before the RCA team meeting. In contrast, phase two explores the knowledge of the RCA members of the process, from the first team meeting to the completion of the final report.

**Table 1:** Overview of data and analysis methods used in the case study

Data and relevant documents	Analysis method	Description
Individual in-depth interviews with all participants in the RCA team	Qualitative thematic analysis	The informants were interviewed sequentially during the RCA process, before starting and after implementation, with a direct focus on how they experienced the RCA process
The written mandate from the process owner	Document analysis	The written document from the process owner specifies the answers the hospital department manager wants to receive in implementing the RCA process. The document was examined, and the content was interpreted, analyzed, and incorporated into the interviews
Procedures in the clinic	Document analysis	Procedures in the clinic that the RCA team used during the implementation. Compared previous approaches with new procedures that were changed in connection with the RCA process
Final RCA report	Document analysis	Unofficial report of the RCA process, which is delivered back to the process owner, written by the CQO in collaboration with the other team members
Healthcare legislation and legal texts	Document analysis	Excerpts from legal texts that describe the duties of healthcare organizations and health personnel related to this case
Risk and incident analysis – Handbook for the health service	Document analysis	National guideline of the health service, which provides information on the methodology

### Setting and sample

In this case study, the inclusion criteria involved purposeful sampling of a case that was directly pertinent to the research question. Purposeful sampling was employed to identify and examine a case that could provide insights, depth, and relevance to our research. The sampling criteria were specifically designed to include RCA teams conducting RCA processes following a sentinel event. A hospital reached out to us when they were preparing to carry out an RCA process following a sentinel event. As a result, we conducted our study at the first hospital that contacted us.

The case study was conducted at a medium sized Norwegian hospital (approx. 6,000 employees), where RCA was implemented in 2016 and used 2–4 times per year. Specifically, we followed healthcare professionals in the RCA team as they carried out an RCA process. The Chief Quality Officer (CQO) advised on who should be recruited for the study. The RCA team consisted of six participants: three from the quality management department (the CQO, a quality adviser in the clinic, and a specialist medical manager) and three team members who were physicians and experts in their respective fields (henceforth referred to as “medical experts”) (Table 2). The first author interviewed the health personnel who participated in the RCA team. Participants had various experiences, providing different perspectives and rich data sources [32].

**Table 2:**
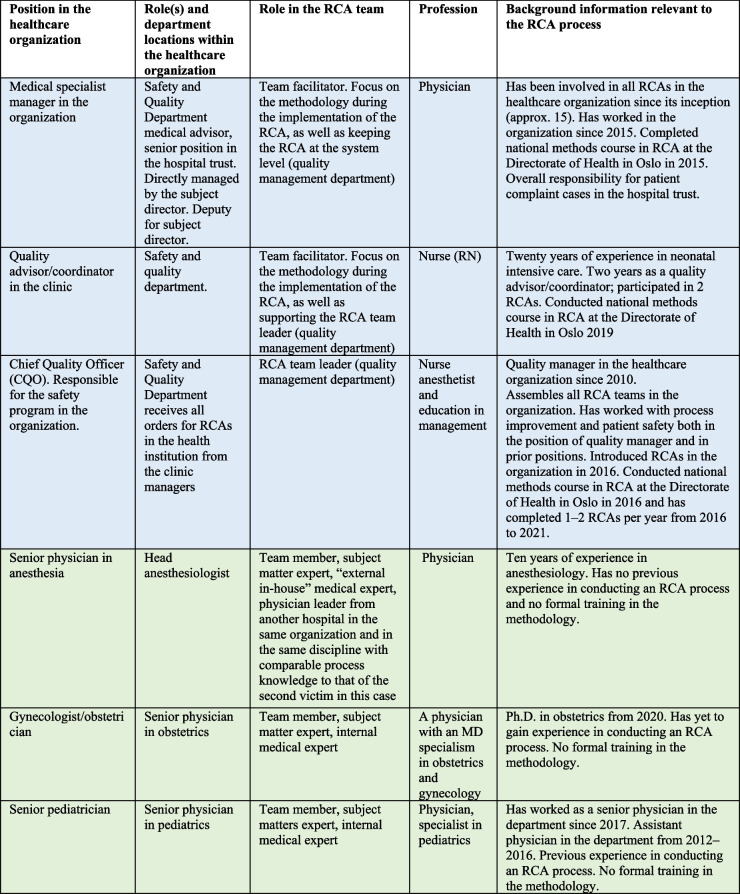
Characteristics of participants included in the interdisciplinary RCA team

Blue shading indicates team members from the quality management department. Green indicates internal/external medical expertise

### Data collection

The first author began the recruitment process in January 2021 by contacting Norwegian hospitals to gain information about RCA teams that had experience using the RCA methodology following the Norwegian national guidelines. Based on the information they provided, four hospitals were recruited. They received an email with a study description, an invitation to participate, a consent form, and contact information for both the first and the last authors. One hospital agreed to contribute by allowing the research team to interview staff (with voluntary consent) after a sentinel event had occurred in their organization. Data collection began in May 2021 and lasted through August 2021. Twelve individual interviews were conducted with six participants (Table 2). Interviews were carried out digitally because of the COVID-19 pandemic and related restrictions. The same researcher conducted all interviews, which lasted approximately 1.5 h each and were audio-recorded and transcribed.

### The process of thematic coding

To analyze the interviews, we used reflexive thematic analysis [33]. Thematic analysis is a method for developing, analyzing, and interpreting patterns across a qualitative dataset, which involves systematic data coding to develop themes [33]. The analytic process was performed based on six phases: (1) dataset familiarization; (2) data coding; (3) initial theme generation; (4) theme development and review; (5) refining, defining, and naming themes; and (6) writing up.

The first author transcribed the interviews. The first phase (1) of the inductive thematic analysis involved the entire research team reading and actively re-reading the data material, searching for meanings and patterns to make sense of the data. An inductive, data-driven approach helped us identify themes strongly related to the data without trying to fit it into a pre-existing coding frame or an analytic preconception [34]. The second phase (2) consisted of coding conducted separately by the first and last authors (Table 3). Initial codes were later reviewed and compared to capture explicitly stated ideas relevant to answering our research questions. We focused on capturing specific and complex concepts and explicit meanings pertinent to our research question in a systematic interpretive semantic approach (participant-driven). The data were analyzed in NVivo version 20/1.3 (for Windows). In the third phase (3), we identified shared patterns of meaning across the dataset, and the research team constructed themes. In the fourth phase (4), we reviewed the themes and considered their relationship to existing knowledge. The fifth phase (5) involved writing a brief synopsis of each theme. In the sixth phase (6), we aimed to weave together our analytic narrative and produce the Introduction, Methodology, and Conclusion sections.

**Table 3:** Examples of the coding process in the interview dataset

Data extract	Code	Theme	Subtheme
“We aim to do this as well, that we teach the clinic how to think, that they can become better at thinking in those directions when they must work with deviations too! Moreover, maybe carry out mini analyses internally in the clinic so that we, in a way, connect the clinicians in our teams and quality advisors from the clinics and so on, that we train them in the RCA method so that they can use it themselves to a greater extent.”	Quality manager’s ambitions Make use of RCA in the clinic	The management system	Quality management department’s role in the RCA process
“When the police come in with a different starting point to us, it interferes with our analysis. It disrupts the relationship of trust with those who are supposed to contribute. At the same time, I understand that they must carry out their investigations….”	The role of the police interferes with the RCA process Understanding of police work	External and internal assessment	Police work interrupting the RCA process

### Ethical approval

Regional Committees for Medical and Health Research Ethics (REK) (reference #195549) considered that the project could be carried out and published without approval from REK based on the understanding that the project does not fall within the scope of medical and health research as defined in the Health Research Act, but instead qualifies as a quality improvement project. Except for the members of the RCA team, no personally identifiable data was collected during the project. The handling of personal information in the project was conferred with The Norwegian Centre for Research Data (NSD) (project number 562024) according to requirements in the act in relation to personal information and GDPR. Additionally, all participants gave their informed consent. This project constituted a quality improvement initiative that granted the hospital the authority to make decisions regarding the utilization of essential patient-related information, following the provisions of both Sect. 26 and Sect. 29c of the Norwegian Act relating to health personnel. Participants shared sensitive information, not only about themselves but also about third parties. The research team discussed how we could develop elaborate, context-sensitive strategies to preserve the richness of the interview material where possible while protecting participants. To protect the participants, the patient, the patient’s relatives, and third parties from ethical issues that might arise from research data being published, we also obtained permission from the CQO. Internal documents that the organization used during the implementation of the RCA analysis are exempt from public access. To balance two competing priorities—maximizing the protection of participants’ identities and maintaining the value and integrity of the data [35]—we chose to anonymize some of the quotations from individual participants and restrict readers’ access to the unofficial internal documents.

## Results

**Fig. 2:**
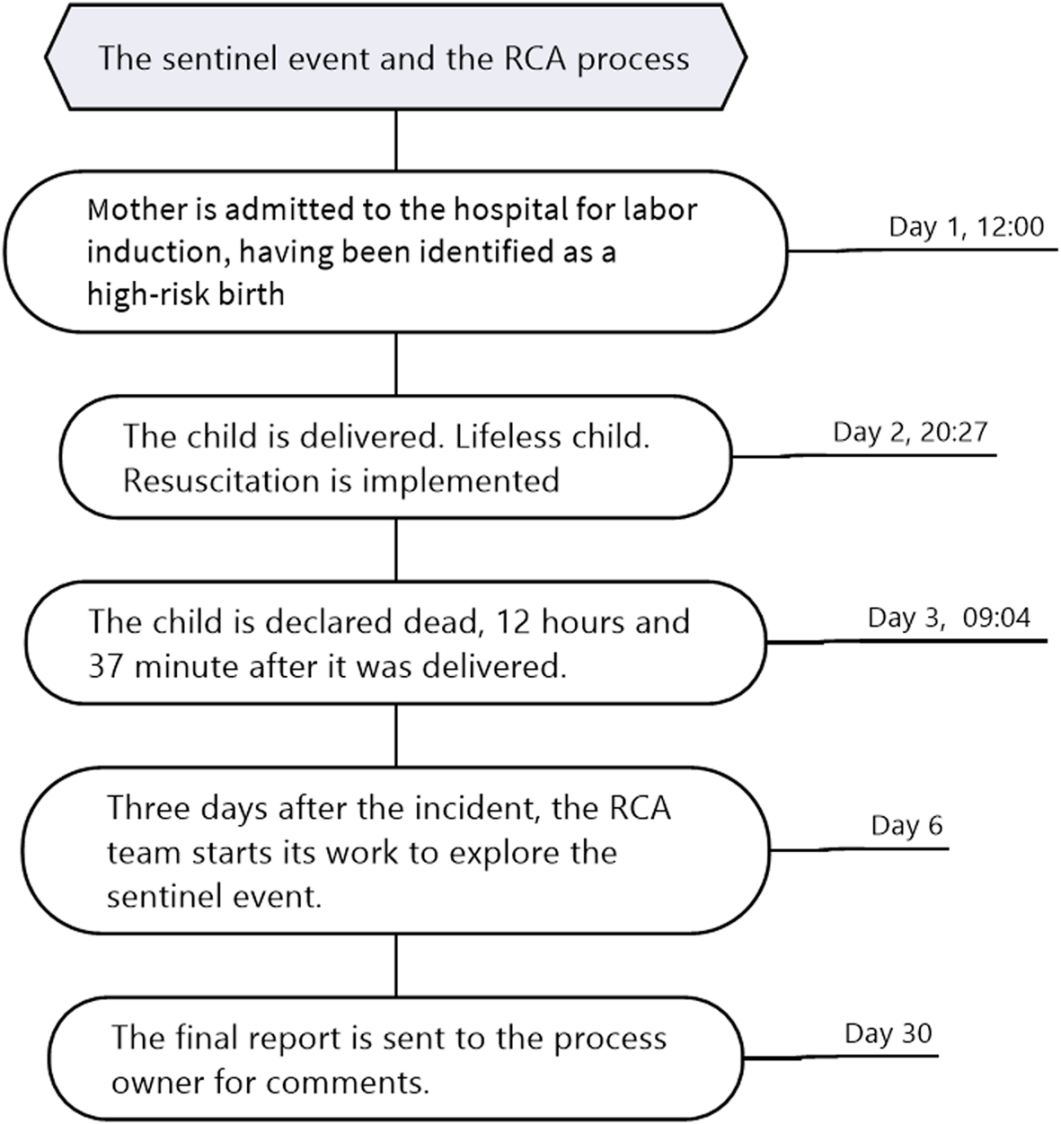
The course of the sentinel event and the subsequent RCA process

The results are presented first with a case description, illustrated by a timeline of the sentinel event and the subsequent RCA process (Fig. 2), and then with a description of three main themes and sub-themes we identified in the data. The first theme, referred to as “the management system,” outlines how the hospital management system manages sentinel events. The second theme, which we named “external and internal assessment,” focuses on how the RCA team perceived the role of “externals” and “internals” in evaluating the incident. Finally, the third theme, “being a team member,” describes how team members experienced carrying out an RCA process.

### Case description (The RCA team’s summary from the final report)

A woman giving birth [number of births], identified as a high-risk birth due to known gestational diabetes and a previous cesarean section, is admitted for labor induction at week [weeks of pregnancy]. She receives a birth epidural assessed to have little effect during the birth. A new epidural is placed, but the woman gets high spinal/blockage symptoms after this admission. The anesthesia takes over with subsequent paralysis of the muscles, leading to breathing difficulties and signs of hypoxia. An emergency cesarean alarm is triggered, and the baby is delivered quickly but emerges pale and lifeless. Life-saving treatment is carried out on the baby. The baby’s heart is successfully started; however, the child has symptoms of extensive damage compatible with oxygen deprivation. The treatment ends the next day. The baby is declared dead around 12 h after birth.

### Theme: The management system

The term “healthcare management system” encompasses the collection of policies, procedures, and practices that govern the decision-making processes and operations within a healthcare organization. In Norway, the management system is primarily described in the Norwegian RCA guidelines [4] and regulated by different laws and regulations [24, 36, 37]. It outlines the requirements for conducting an RCA process, stating that “the hospital’s management system must have procedures in place for assigning the task of initiating an RCA (as shown in the process owner Fig. 1). The procedures and descriptions of responsibilities must be well-known throughout the organization” [4]. The management system’s activities describe the processes and methods employed to plan, implement, evaluate, and correct the organization (hospital) to ensure compliance with healthcare legislation.

### Quality management department’s role in the RCA process

The hospital trust, considered of medium size in Norway and overseeing four hospitals, had experienced several RCAs within a brief period concerning sentinel events during childbirth. Due to the gravity of the situation, the client manager was asked whether the quality department had the necessary resources and methodological expertise to conduct the RCA independently within the clinic. The Chief Quality Officer (CQO) acknowledged that they needed additional methodological expertise to carry out the RCA properly. The quality management department was brought in to provide expertise on the RCA method to ensure a methodical, correct, and professional approach. At the same time, employees working closely with patients were included in the RCA process. This was expected to facilitate knowledge-sharing and keep the process at a system level.

Members of the quality management department reported that their participation in the RCA process led to new insights regarding the importance for healthcare personnel of adequate rest, the need for more education, and the need to refresh the skills of experienced healthcare workers. They stated that RCA was performed on the most sentinel events in the hospitals, and it was vital that the management took responsibility by carrying out an RCA process when a sentinel event occurred. The CQO worked purposefully to introduce and implement the RCA methodology in the organization and stated that RCA had improved its foundation over the past years. However, the CQO also expressed concern about the low number of RCAs conducted in the organization. “I have sometimes wondered why we carry out as few RCAs as we do. I believe that there are several incidents that we should have investigated, and I am not sure whether it is from the management’s side; it may well be that there is skepticism from employees or a lack of knowledge and understanding when serious things happen and that there is a need for a systematic review to learn.” It was pointed out that they previously had insufficient tools to promote learning after a sentinel event and that RCA was not integrated into everyday thinking. Recognizing the significant learning value of sentinel events and the importance of conducting systematic reviews did not occur naturally. The quality management department realized they needed more general knowledge of the methodology to achieve psychological security in the organization. They pointed out that the current management system was not designed adequately to care for employees after a crisis and that RCA initiation could be an additional psychological burden. They also recognized the need for better follow-up of employees after an RCA process.

The quality management department played an active role in implementing national quality strategies and quality improvement requirements within the organization. However, there were different opinions within the organization on how the quality management department works with quality improvement. The quality management department had experienced resistance from some employees. Some employees were given feedback that they needed to better understand the quality management department’s role. In contrast, those who collaborated with them gained knowledge concerning their support function within the organization and quality improvement. The department appointed a contact person for each clinic to improve collaboration and communicate information about quality improvement tasks. However, some employees perceived the department as an ordering unit rather than a facilitator due to role confusion.

The CQO considered the quality management department a significant asset in implementing the RCA method. Conducting an RCA process was very resource-intensive for the departments involved, and it took work resources from everyday clinical life. It was pointed out that it was not easy for a clinic to carry out an RCA without support from the quality management department. The RCA methodology was considered challenging to implement correctly and not sustainable in the long run. There was a clear rationale for considering RCA unsustainable to carry out following all undesirable events. Therefore, a shortened version of the RCA process was needed, and the team requested national guidelines to support this challenge.

### Scattered documentation

The medical experts in the RCA team pointed out that finding all the necessary documentation related to the incident was challenging because the documentation was scattered across different systems and platforms. Despite having ample time to locate the documentation before the RCA team meeting, the medical experts found it challenging to gather the necessary information. This also posed problems for daily clinical follow-up, as information could be missed due to the scattered documentation. “The documentation was spread over many different platforms—so it was difficult to find, which is unfortunate! Both regarding inspections and everyday clinical life where information may be missed.” Furthermore, those outside the organization had difficulty understanding the logic behind each medical journal’s tradition and culture for documentation practices. Therefore, it was considered important to put together an overarching RCA team that works daily with the various work processes and could understand the whole process. The analysis team discovered considerable variation in documentation practices within the organization, making comparing documentation practices in different fields difficult. This variation could potentially threaten patient safety and heighten the need for standardization.

### Theme: External and internal assessment

This theme focuses on how the RCA team viewed the role of “externals” and “internals” in assessing the incident. “Externals” refers to entities such as the police or external medical experts from other hospitals involved in the RCA process. They are often perceived as more neutral and impartial, which can benefit a particular analysis. The document analysis revealed that the Norwegian guidelines only partially describe the role of externals. The guidelines require that the analysis team must be multidisciplinary, that all professional groups and subject areas affected by the analysis must be represented, that a physician must be part of the analysis team, and that it is essential to include people who can add an “outside perspective” [4]. The guidelines indicate that an inappropriate size or composition may omit critical perspectives and impair the report’s quality and legitimacy.

### Composition of the RCA team

It emerged in the interviews that the RCA team was satisfied with the composition of the team. The RCA leader (QCO) decides the team composition in collaboration with the analysis team members and the organization managers. The RCA team comprised six members (as shown in Table 2); half of these were quality management department employees, while the other half consisted of medical experts from various departments. The team’s composition represented different aspects of the sentinel event. However, the team recognized that involving other professional groups, especially medical experts without close involvement in the incident, would have been beneficial. The “external- in-house” medical expert pointed out that other professionals, such as midwives, could have provided critical perspectives: “It might have been more appropriate to include other professional groups, such as midwives, to represent a more holistic composition of different professional groups.” The team members emphasized that working together during the process was seen as constructive, and they were able to come to a consensus.

The management department employees found the process challenging because team members had varying levels of experience with applying RCA. The same employees had participated in national training in the methodology and had conducted multiple analyses within the organization. All medical experts were familiar with the method through previous incidents in their clinics, but this was their first time carrying out a complete RCA process. The team members expressed that employees’ varying levels of experience with the RCA methodology could pose challenges in identifying root causes. Some team members suggested that increasing familiarity with the RCA method would be beneficial for fully understanding the RCA process within the organization. The team acknowledged that variations in experience with the RCA method could affect the quality of recommendations.

### Police work interrupting the RCA process

Physicians working in Norwegian healthcare organizations are required to report to the police if an unnatural death is suspected [38]. The purpose of this requirement is to inform the authorities that a death has occurred, and an investigation can be initiated if the death appears unnatural. In the RCA team’s mandate, one of the issues was to investigate whether the clinic had appropriate routines for notifying the police in such cases. However, due to internal disagreements, the sentinel event was reported to the police belatedly, leading to a delay in the RCA process. The National Criminal Investigation Service (NCIS) investigated the case with local police to provide specialist expert support. This delayed the interview process in the RCA, as the police and NCIS had to conduct their interrogations before the actual analysis could be initiated.

Several members of the RCA team describe the police’s involvement in the case as disruptive to the internal RCA process. The police also had a very different approach from the RCA team and were perceived as brusque, foreign, and suspicious. “At first, they stepped in, and they were a little brusque, and they came in here and acted strange. They were rough, perhaps because they are used to communicating with people with something to hide!” Some employees felt that the incident was perceived as more serious when the police got involved. The police had turned up at the hospital for a crime scene investigation without reporting in advance. They expected this would be hidden from employees if evidence was tampered with. “Now we are standing outside—can you come and lock us in? Do not tell anyone we are here. In other words, so that they hide something before we come up.” The police then went in and seized evidence inside the crime scene.

One of the medical experts who had worked in other parts of the country gave feedback that it was common practice to report to the police in other healthcare organizations and noted that this element was handled entirely differently in this organization. The RCA team concluded that their organization had no culture and little knowledge and practice of reporting unnatural deaths to the police, which highlighted the need to work on implementation at both the clinic and organization levels.

### Using colleagues to provide external medical expertise is seen as challenging for the process and the participants

In the RCA team’s mandate, it was requested by the process owner that a representative of the anesthetists should be part of the analysis team. The RCA team decided to extend this to include all specialist fields involved in the sentinel event. Previous RCA teams had brought medical expertise from outside the organization to offer an external perspective. The challenge in this RCA was that none of the other three hospitals in the organization had a neonatal ward and the expertise this provides. The RCA leader discussed with respective managers whether external medical expertise should be brought in. However, for this RCA, the decision was made to bring medical expertise from within the organization. They acknowledged that obtaining professional knowledge from within the hospital trust was not optimal, as other physicians could consider this decision in the organization as a mistake. At the same time, it was concluded by the RCA leader and the management that they could make use of medical experts who were not directly involved in the sentinel event. Therefore, they weighed the advantages and disadvantages and considered it prudent to use internal medical expertise in the RCA team. It was emphasized that the decision had been made because this was not an external inspection. They believed they had made the right decision by ensuring that the “external in-house” member had not been directly involved in the sentinel event.

RCA team members disagreed about using internal medical expertise, especially regarding colleagues considered “second victims” in the incident. The “external in-house” medical expertise pointed out that external medical specialists could provide a new perspective and make it easier to identify negative work patterns. Spending a long time in the same environment can lead one to become unaware of work habits. Team members suggested that the validity of this RCA could be improved by including external medical expertise and seeking expertise from outside their organization. Evaluating one's colleagues in a relatively small environment was considered unconstructive in the implementation itself. The “external in-house” medical expert expressed that it would be emotionally challenging for the other members to evaluate the actions of their colleagues in the severe event. “I believe I was deeply emotionally detached when it came to those involved, their emotions, and everything related. My presence was primarily focused on the medical aspect of it.” The “external in-house” expert pointed out that internal medical experts should receive support and recognition for their work in the implementation. Still, a consequence could be that internal medical expertise could be affected by the emotional involvement of their colleagues.

The quality management department acknowledged that they had received feedback from their employees in the past that it was challenging to evaluate colleagues while also carrying out an RCA. “It is tough to evaluate colleagues in the way that we do. It is a heavy burden for those in the situations, also in the time afterward, because there are tough assessments and things that happen in ‘a blink of a moment’ that are analyzed thoroughly. It is demanding to be a colleague afterward. Because it hurts, it is a defeat. You have been involved in something challenging, and then it must be assessed afterward, and it is easier if you do not have a relationship afterward.” They pointed out that assessing a situation was easier when there was no prior relationship with those involved.

### Theme: Being an RCA team member

The Norwegian RCA guidelines explicitly stipulate that “team members must strive to work neutrally with no other interests than increasing patient safety” [4]. This theme is presented with detailed depictions of team members’ experience with the RCA process and the self-awareness they developed during its implementation.

### Role challenges

The quality management department remarked that it was challenging for managers to understand the methodology or familiarize themselves well enough with it. They emphasized the need for increased communication with managers to ensure they understand the RCA process, their role, and their responsibility for employee follow-up after a sentinel event. One team member coordinated with the police and found it challenging to reassure healthcare staff involved in the RCA process while gathering information for the police and acting as a liaison within the organization. Having two different roles could confuse the purpose of the RCA, leading to insecurity among employees. The team members noted that employees might fear that the RCA could result in disciplinary sanctions and stressed the need to clarify their role as internal investigators. They acknowledged that employees and managers might struggle to distinguish between these roles and emphasized that it was crucial to separate external supervision from the internal investigation. However, it was difficult to determine how employees perceived these roles. For this reason, they emphasized the importance of informing employees about the two distinct roles and clarified this explicitly during interviews.

### Ambivalence about being an RCA team member

The roles and motivations of the RCA team members were diverse and complex, with some expressing ambivalence about their participation. On the one hand, they felt a sense of obligation towards the families who had experienced a loss and their colleagues who were also impacted. “I think we owe it to those who have experienced losing their child, as well as health personnel who have experienced this.” They valued the educational aspect of the process and appreciated the chance to work with other experts and learn from resource personnel within the organization. They believed that carefully examining sentinel events, particularly those with significant consequences, was critical. Nonetheless, they found it challenging to scrutinize their colleagues, especially when they discovered mistakes. Some members initially hesitated to participate but felt compelled because the professional pool was too small to make it practical to choose other team members. Some medical experts agreed to join the RCA because they were confident that they would not uncover errors made by their colleagues.

The RCA process was deemed arduous and time-consuming, taking valuable time from team members' already hectic clinical work, and leading to heightened stress levels. It also meant sacrificing holidays and leisure time. Performing an RCA was equivalent to taking on additional work on top of the demands already placed on clinicians. This required downgrading or delegating clinical work to others, which was challenging in an already stressful work environment. Previous RCA processes in the organization had indicated that healthcare personnel should have acted differently by adhering to best practices. The members of the quality management department faced challenges when the RCA process revealed that healthcare personnel had made mistakes. Even when the RCA process showed that health personnel had worked under suboptimal conditions, it was difficult for the RCA team members to reconcile their emotions when they realized their colleagues had not performed their duties correctly. Despite this, all RCA team members showed engagement and held positive discussions during the analysis process. However, reading about what had happened to the child and mother in medical journals and interviewing second victims was a demanding and painful experience. Some in the team found this case burdensome and challenging, especially those with close colleagues in the clinic who had dealt with the severe event. Although some team members found the process exciting and instructive, they did not want it to cause additional stress for their colleagues. Medical experts found it exciting to delve deeply into the literature related to the incident and the procedures involved. However, they also expressed that the process was emotionally straining and was not adequately followed up.

## Discussion

Our study allowed us to explore an RCA team's experiences during the early and late stages of an RCA process. We identified three main themes and several sub-themes related to the RCA process (see Results). Further, we compared our findings with the recommendations in the Norwegian RCA guidelines [4]. Our study adds to a growing body of research on the challenges of using RCA [19, 39, 40]. In general, the literature shows that previous studies have also identified implementation barriers in the RCA process related to (1) technical/methodical barriers [11, 41–43]; (2) organizational barriers [43–48]; and (3) individual (human) factors [49]. Overall, this literature points to significant challenges in the RCA process that center on translating RCA methods into practice and removing barriers to improve healthcare quality and effectiveness.

The results indicate that the RCA team faced various challenges during the RCA process, including the difficulty of being neutral, role challenges, ambivalence about being an RCA team member, and the additional burden on time and resources. Although the analysis process was demanding, all members expressed engagement in participating in the RCA team. Further, the study findings indicate that there can be significant challenges in applying the Norwegian RCA guidelines as laid out by health authorities. This case shows that the management system presupposes clear descriptions of responsibilities, and easily accessible information, which can be challenging to achieve in this complex environment. At the same time, we found that challenges related to medical documentation (scattered documentation), the role of the police, and the ideal of neutrality in the assessment of colleagues (external and internal assessment) indicate the need to problematize certain recommendations in the Norwegian RCA guidelines.

Experts suggest that bridging the gap between understanding and implementing systems thinking requires more RCA training and event analysis methods that promote systems thinking [19, 50]. Our case study reveals the critical role of the quality management department in the RCA process, both during implementation and as methodological support throughout the process. The department had permanent members who participated in the RCA process and determined who should lead it. We consider that this approach could benefit from learning about and developing systems thinking knowledge and methodology within the organization. However, no data can confirm whether this outcome will be achieved. Nicolini, Waring, and Mangis [12, 42] highlight various barriers to learning from patient safety incidents within the UK healthcare systems. These obstacles encompass leadership challenges within the investigation team, inefficient information analysis processes, inadequate staff participation, time and resource limitations, competing priorities, insufficient change expertise within investigation teams, conflicting perceptions of the nature of the problems, and a lack of organization-wide sharing of localized learning. Complex issues are sometimes disregarded due to the perceived difficulty of resolving them. Additionally, producing a well-crafted RCA report is often considered a desirable end goal in and of itself [12]. Hospitals face many demands, which may cause some hospitals to treat RCAs as a "checkbox exercise" to meet accreditation requirements rather than as an opportunity to identify areas where fundamental changes are needed and improve the hospital's safety culture. This may challenge the management's priorities regarding which measures are sustainable over time. Since the RCA process requires numerous resources from an already resource-stretched organization, initiating an RCA process may create a methodological internal barrier.

In healthcare, quality management refers to the administration of systems design, policies, and processes that minimize or even eliminate harm while optimizing patient care and outcomes [51]. The quality management department's understanding of how the system in the organization works under pressure seems crucial in this case. Although they acknowledged the need to gain more general knowledge of the methodology for achieving psychological security in the organization, they had made progress in admitting the challenge of following up with employees after the sentinel event. Actionable measures were implemented to increase their knowledge of the methodology. However, the quality management department's expertise in quality development in the organization might need to be recognized by others in the organization.

Members of the RCA team expressed that evaluating the case and their colleagues was emotionally challenging. Therefore, being neutral and having no other interest besides increasing patient safety was difficult. This reality deviates from the idealistic approach in the Norwegian RCA guidelines, which requires the RCA process to be carried out with neutrality on the part of the participants. Colleagues of the RCA team had experienced both being the second victim in the case and being inflicted with a new trauma when the RCA process was initiated. Police investigations and media exposure also made it challenging to carry out the RCA in the organization. We argue that expecting neutrality in an RCA process is unrealistic because the issues being investigated involve people with emotions. In some cases, people involved may have a vested interest in the outcome of the analysis. Additionally, the emotional impact of the incident may make it difficult for individuals to remain detached and impartial. All these factors can make maintaining neutrality throughout the RCA process demanding, with a risk of inaccurate analysis.

In previous studies, RCA has been shown to present several challenges associated with forming and leading the investigation team, gathering and analyzing supporting evidence, and formulating and implementing service improvements [42]. In this case, the way the team was put together, with both permanent team members and medical experts from each discipline, meant they could see unintended patterns and barriers and provide a comprehensive overview of the organization’s system challenges. This case indicates that management and quality management departments’ involvement in the RCA process was crucial for employees' development and views on the methodology. The study gave us the impression that the RCA methodology had only partially taken root in the organization.

Our results also revealed internal disagreement concerning which deaths should be reported to the police. The organization had no established routines for reporting cases like this to the police. The involvement of the police leads to fear of external supervision. Employees and members of the RCA team experienced that the police investigation disrupted the RCA process and alienated them. Part of the explanation could be that this was experienced as a new way of handling death. At the same time, it was pointed out by the RCA team that one had to be able to see the police involvement from the viewpoint of the parents who had unexpectedly lost their child and who wanted answers as to why their child had died. This consideration was challenging to acknowledge for the employees and some RCA members when the police started their external assessment of the event.

### Implications for practice

Hopefully, this article contributes to a better insight into the complexity of the RCA process. The RCA process is not a prescribed method; each approach will vary [18]. Each sentinel event, organization, and context are unique and determine how this tool is used in practice. The Norwegian RCA guidelines stress the importance of precise routines and responsibilities. Should the guidelines be more flexible about discretionary assessments in some areas and more clarifying in others? Clinician participation in RCA is crucial as these initiatives recognize and address essential patient care aspects, but our study shows there are barriers to clinician participation. It also shows that the clinicians requested an abbreviated national version of the guidelines, and that the method is resource-intensive and demands a lot from staff involved in the process. Therefore, one can question whether a full-scale RCA method is sustainable in an already resource-stretched organization.

### Study strengths and limitations

This study has two main strengths. The first is that the first author interviewed participants in the RCA process twice, allowing for an in-depth exploration of themes. Secondly, we used a case study approach to gain a detailed understanding of the research subject. This approach allowed for a thorough investigation of the specific case under examination. However, there are also limitations. This study exclusively concentrated on the RCA process at the hospital level, based on a single case. As a result, the findings may not be readily transferable to different settings or hospitals. It is imperative to exercise caution when attempting to extrapolate these findings beyond the hospital context. The researchers had no control over the selection of participants in the study, which may have influenced the composition of the RCA team and the process itself. The study focused on the RCA process at the hospital level only and may not apply to other settings. While this focus provides valuable insights into the teams’ perspective, it may not encompass the broader context and stakeholders involved in the RCA process. Nor have we interviewed the mother and next of kin due to ethical challenges. This limitation may have left a gap in the overall understanding of the RCA process, particularly from the perspective of those directly affected. We have limited the study to explore the experiences of the RCA team. Additionally, the researchers did not directly observe the team's work; doing so could have provided insider views and subjective data [31]. Finally, the study’s findings are based on a single case, and consequently, the generalizability of these findings should be interpreted in the context of the study’s design.

### Future research and development

Future development could include a national register for RCA documentation to track trends and promote learning. The lack of official documentation of the RCA process and final report means that only a few people have insight into the process. Making the final report official and anonymous could increase transparency and trust within hospitals and reassure patients and their families. Interviews are critical for understanding an incident’s cause, but recall bias often delays or affects them. Gathering information from all relevant parties while the incident is still fresh is crucial for successful documentation. The involvement of police in the RCA process can delay interviews and create a more serious atmosphere, which can affect the process. To improve the RCA process, individual professionals should increase awareness and efforts to achieve documentation by providing adequate resources, clear mission statements, and coherent policies. Involving permanent members from the quality and safety department staff in several RCA processes can provide a better basis for comparison. Finally, the Norwegian Board of Health Supervision emphasizes collecting information from all relevant parties, including patients and their families.

## Conclusion

This study is one of few studies that have explored RCA team experiences during an RCA process and whether there is adherence to the Norwegian RCA method. The results may inspire healthcare organizations, health authorities, quality and patient officers, and RCA teams to improve the RCA guidelines further and learn from this case. This study has shed light on three critical RCA team experiences: Firstly, the intricacies of the healthcare management system, particularly the role played by the quality management department, underscore the need for seamless integration of RCA into everyday practices. The integration is vital for enhanced support and guidance to employees during crises.

Secondly, the delicate balance between internal and external assessments in the RCA process highlights the value of embracing diverse perspectives and expertise. This inclusivity is essential to obtain a comprehensive understanding of sentinel events.

Lastly, the challenges and ambivalence encountered by RCA team members as they evaluate their colleagues and navigate their roles emphasize the need for clearer communication and robust support mechanisms.

These experiences have practical implications for healthcare organizations, RCA team, and professionals. It is, therefore, necessary to streamline the management system, promote a culture of continuous learning and support, and ensure that RCAs benefit from both internal and external insights. To address these issues, we recommend a renewed focus on education and training, improved collaboration between internal and external stakeholders, and more robust support system for RCA team members.

In the broader context of patient safety discourse, these experiences emphasize the need for a proactive and holistic approach to quality improvement. By addressing these key experiences, healthcare organizations can move closer to a safety culture where patient well-being remains paramount. Ultimately, being aware of and acting upon these critical RCA team experiences can hopefully lead to safer and more effective healthcare practices for all.

## Data Availability

The data generated and analyzed in the current study are not publicly available due to Norwegian privacy legislation and the form signed by the participants about the study`s privacy. The data generated are available from the corresponding author on reasonable request.
